# Transitioning to a high renewable net-zero power generation system in Malaysia

**DOI:** 10.1098/rsta.2021.0132

**Published:** 2022-04-18

**Authors:** Hoy-Yen Chan, Kamaruzzaman Sopian

**Affiliations:** ^1^ Academy of Sciences Malaysia, Menara Matrade, Tingkat 20, Sayap Barat, Jalan Sultan Haji Ahmad Shah, 50480 Kuala Lumpur, Malaysia; ^2^ Invite Green Consultancy, Lintang Pantai Jerjak 3, Gelugor 11700, Penang, Malaysia; ^3^ Solar Energy Research Institute, Universiti Kebangsaan Malaysia, Bangi 43600, Selangor, Malaysia

**Keywords:** net-zero, carbon neutrality, roadmap development, carbon-free energy, backcasting, generation mix

## Abstract

Malaysia is a net importer of coal, petroleum products and piped natural gas. Moreover, its primary energy supply is dominated by fossil fuels, at about 93% in total, with coal and natural gas constituting the highest shares in electricity generation. Thus, there is need for Malaysia to take swift action in transitioning to a high renewable energy system for long-term sustainability and meeting its climate action commitment under the Paris Agreement. A net-zero emissions vision guided by a roadmap may effectively motivate and catalyse carbon-free energy deployments. In this paper, we revisit the carbon-free energy roadmap that was developed in 2015 and compare it with the current generation development plan to identify the gaps between them. We argue that the roadmap is still relevant to the net-zero emissions vision; however, we have also identified gaps that merit further research and improvement. The identified gaps mainly relate to more recent data, along with technology and policy developments. Accordingly, we put forward potential research suggestions to bridge these gaps for future development of a roadmap that would assist Malaysia in shaping a long-term plan towards realizing a high renewable net-zero power generation system.

This article is part of the theme issue ‘Developing resilient energy systems’.

## 1. Introduction

Malaysia is one of the world's largest oil and gas (O&G) producers, ranked 26th and 13th, respectively [1]; in 2020, mining and quarrying of O&G contributed about 6.8% of its gross domestic product (GDP) [2]. Nonetheless, this contribution has been declining since 2018, even before being hit by the impact of the COVID-19 pandemic; in 2020, the GDP contribution of this sector contracted by nearly 11% relative to 2019 [2]. Furthermore, Malaysia has been a net importer of petroleum products and piped natural gas since 2010 and 2005, respectively [3], and coal demand has been increasing, with net imports annually growing by about 9.3% on average since 2015. The power sector is Malaysia's largest consumer of natural gas and coal; in 2018, its primary energy supply was dominated by fossil fuels, at about 93% in total, with O&G individually accounting for 71%, coal for 22% and renewables for 7% [3]. Thus, the energy sector in Malaysia will not be sustainable in the long term and will require increasing imports of fossil fuels to meet growing domestic energy demand. Together with the contraction of O&G contributions to the country's economy, we justify the need for Malaysia to take swift action in transitioning to a high renewable energy (RE) system (table 1).

**Table 1.** Nomenclature.

ACF Roadmap	ASM Carbon-Free Energy Roadmap
ASM	Academy of Sciences Malaysia
BAU	business as usual
CO_2_	carbon dioxide
EE	energy efficiency
EV	electric vehicle
FiT	feed-in tariff
GDP	gross domestic product
GHG	greenhouse gas
GtCO2	gigatonnes of carbon dioxide
GWh	gigawatt-hour
IPCC	Intergovernmental Panel on Climate Change
IRENA	International Renewable Energy Agency
LCOE	levelized cost of energy
LSS	large-scale solar
MW	megawatt
NEEAP	National Energy Efficiency Action Plan
NEM	net energy metering
NDC	nationally determined contribution
O&G	oil and gas
OTEC	ocean thermal energy conversion
P2P	peer-to-peer
PV	photovoltaic
RE	renewable energy
R&D	research and development
TPES	total primary energy supply
UNFCCC	United Nations Framework Convention on Climate Change
VRE	variable renewable energy

In addition, Malaysia ratified the Paris Agreement, which set a long-term temperature goal, specifically limiting the global average temperature rise relative to pre-industrial levels to well below 2°C (preferably 1.5°C) as of the end of the century [4]. To achieve this, cumulative CO_2_ emissions will need to be kept within a budget and global annual CO_2_ emissions reduced to net-zero (carbon neutrality). According to the Intergovernmental Panel on Climate Change (IPCC), the probability of limiting global warming to 1.5°C is about 66% if carbon neutrality is reached by 2040, or 50% if neutrality is achieved by 2050. For the former scenario, the estimated remaining carbon budget is about 420 GtCO_2_; for the latter, it is about 580 GtCO_2_ [5]. A lower carbon budget means a lesser cumulative amount of CO_2_ is released to the atmosphere, and, as suggested by the IPCC, this allows a higher probability of staying within the temperature threshold of 1.5°C. Hence, more aggressive decarbonization is needed to increase the probability of limiting total global warming. Malaysia committed to a 45% reduction in greenhouse gas (GHG) emissions per GDP by 2030 as its nationally determined contribution (NDC) under the agreement [6]. As the energy sector contributed about 79% of Malaysia's total GHG emissions in 2016, decarbonizing the energy system is critical [7].

Many countries have in recent years further set a net-zero emissions goal as part of their climate policies. As of March 2021, Singapore is the only country in Southeast Asia that has formally expressed an intention to achieve net-zero emissions in the second half of the century [8]. Nevertheless, the Academy of Sciences Malaysia (ASM) established a taskforce as early as 2015 to prepare an advisory report, the ‘Carbon-Free Energy Roadmap for Malaysia (2015–2050)’ (hereafter referred to as the ACF Roadmap) [9]. This was done even before Malaysia's official ratification of the Paris Agreement. The taskforce aimed to (i) identify the major stakeholders involved in carbon-free energy, research and development; (ii) identify the issues that need to be faced in implementing carbon-free energy and (iii) develop a blueprint and roadmap for a carbon-free energy programme in Malaysia, including proper funding and public engagement and awareness programmes. This was the first roadmap that considered CO_2_ emissions from the energy sector and illustrated mitigation impacts quantitatively.

The ACF Roadmap was developed through a series of meetings among the task force committees to gather input and discuss technological and deployment issues. In addition, a workshop was conducted to consult with stakeholders from universities, power sectors, R&D institutions, the finance community, industry and government agencies, incorporating their input on carbon-free energy deployment targets and action plans for meeting those targets [9]. Although approached in a comprehensive manner, the roadmap was developed in 2015 and based on the best available energy data from 2012; it merits re-evaluation in the context of more recent data. In addition, current technology readiness might have changed compared to 2015; in particular, recent years have featured acceleration of technology development and innovation, cost reduction and changes of the policy landscape [10–13]. These can be seen in the recently adopted Generation Development Plan 2020, where the types of renewables and their shares in electricity generation differ from those suggested by the ACF Roadmap. Hence, this paper reviews and discusses the relevance of the Roadmap and gaps in it with respect to present developmental trends of the energy sector in Malaysia. We then conclude the discussion by highlighting future research areas that could strengthen roadmap development for Malaysia to move towards a high RE net-zero power generation system.

## 2. Contemporary relevance of the ACF Roadmap

### (a) Alignment of the ACF Roadmap with the Paris agreement

Setting RE and energy efficiency (EE) targets is crucial in implementing mitigation actions. Nevertheless, the global mean temperature is ultimately determined by the total anthropogenic GHG emissions added to the atmosphere; as such, it is most critical to reduce net emissions through avoidance and removal. Thus, while aspirational RE and EE targets are vital, if the ultimate goal is to combat climate change, these targets need to be associated with an emission target. As the ACF Roadmap aimed to shape the path by which net-zero emissions could be achieved, it was deemed relevant to the Paris Agreement's goal.

### (b) Scope of the ACF Roadmap

Two scenarios were developed under the ACF Roadmap, in which ‘carbon-free energy’ was defined in two distinct respects. The first scenario considered carbon-free energy in the total primary energy supply (TPES). Malaysia's energy sector is heavily dependent on fossil fuels, with only about 7.2% of its primary energy supply being sourced from RE [3]. During roadmap development, the taskforce committees and stakeholders found that it was unrealistic to expect 100% carbon-free energy in the TPES by 2050. Therefore, the taskforce developed the second scenario, which defined carbon-free energy as having net-zero CO_2_ emissions in electricity generation and so narrowed the scope from the TPES to electricity generation. Not only did this scenario set a more achievable goal, but also, based on the most recent GHG inventory [7], electricity generation constitutes the major CO_2_ emissions source in Malaysia. In 2016, electricity and heat production together accounted for 39% of the total CO_2_ emissions; the second largest emissions contributor was road transportation at 21%, the third manufacturing industries and construction at 9% and another 16 sectors contributed the remaining 31% of emissions. The average annual growth rate of GHG emissions from electricity generation between 1990 and 2016 was 7.5% [7]. Thus, a study designed to focus on electricity generation will still be relevant to the present state.

### (c) Backcasting approach in the ACF Roadmap

Forecasting and backcasting are two common approaches used in planning and decision-making. Forecasting projects the present situation into the future based on currently available information such as technology levels and industrial structure. While forecasting provides potential scenarios of future development, these scenarios may not necessarily reflect desirable developments, but rather give images of possibilities [14–16]. By contrast, backcasting starts with defining a vision and then analyses pathways by which to realize it, working backwards from the desired future situation to the present. In short, forecasting projects ‘what’ is likely to happen in the future, while backcasting shows ‘how’ a desired future can be attained [16–20]. Therefore, when planning for long-term and novel goals in complex systems, and especially when seeking a solution for a societal problem like climate change, backcasting might be a more appropriate approach [15–17]. Furthermore, backcasting will outperform forecasting when the problem is the mainspring of the forecast trend and is driven by externalities. That is, marginal adjustment of current development trends is not sufficient to resolve the problem; rather, the solution necessitates a major change or extensive improvements. When backcasting, the desired goal usually takes a long-term perspective of 20–25 years, long enough to allow considerable scope for deliberate choice [15,17,18,20].

All these factors support use of the backcasting approach for the ACF Roadmap and frame the needs of that roadmap in realizing the aspirational net-zero vision. Furthermore, although Malaysia had previously set an emission intensity reduction target as early as 2009 [21], connection to and prioritization of CO_2_ mitigation were absent from energy technology roadmaps. Thus, rather than setting out to confirm present development trends, the ACF Roadmap applied the backcasting approach and integrated with stakeholder analysis to explore net-zero possibilities. The roadmap suggested action plans, including policy reform, required measures and technological breakthroughs, and identified actors who can catalyse and realize the changes necessary for achieving carbon neutrality of the electricity generation mix.

It is worth noting that forecasting, besides providing scenarios for future development, is also important in optimizing and finding efficient solutions [18]. Therefore, for the next net-zero emissions roadmap, forecasting can assist in providing an optimized short-term scenario. Such integration would complement the action plans suggested by backcasting in achieving the desired long-term goal.

## 3. Gaps between the ACF Roadmap and the Generation Development Plan 2020

**Figure 1.**
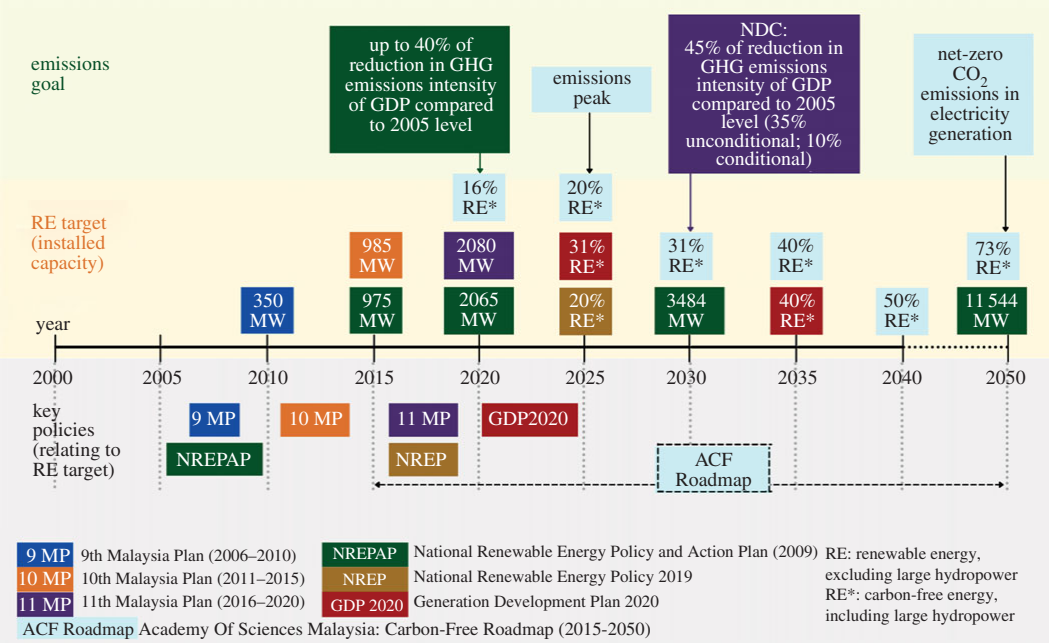
The development of national RE targets in Malaysia. Years associated with policies refer to the period of their adoption while those for RE targets refer to the year in which RE generation was projected to reach the targeted installed capacity; for instance, 350 MW of RE installed capacity by 2010 was the target set under the 9 MP. Policies that set RE targets are represented with distinct colours. Both the ACF Roadmap and the GDP 2020 anticipate 40% RE capacity to be installed by 2035. (Online version in colour.)

Malaysia started to recognize RE as a major fuel in the energy supply in 2001 [22] and has been setting RE targets since 2006. Figure 1 shows the development of RE targets under national policies and plans from 2006 to 2020. In 2009, Malaysia established the National Renewable Energy Policy and Action Plan [21] and the National Policy on Climate Change [23]. The former included RE targets with CO_2_ emissions avoidance, while the latter recognized the roles of RE and EE in achieving climate-resilient development. In the same year, Malaysia announced its first voluntary emissions reduction target at the Copenhagen Climate Change Conference, aiming to reduce the GHG emissions intensity of its GDP by up to 40% relative to 2005 levels by 2020. The ACF Roadmap was the first energy roadmap that considered emission mitigation impacts of the generation mix.

The net-zero vision has only recently gained attention from policy-makers, likely due to global development trends and higher awareness of climate-change issues. However, Malaysia's prioritization of mitigation actions in the net-zero vision has not yet been clearly reflected in the energy policies and targets. The RE target has been the key indicator in measuring the sustainability of the energy sector. In 2018, the share of RE in total installed capacity was 22.2%, with hydro, solar, biomass and biogas accounting for 18.1%, 2.3%, 1.6% and 0.2%, respectively. As of this writing, the most recent RE targets are 31% of total installed capacity by 2025 and 40% by 2035; these were endorsed in the Generation Development Plan 2020 [24]. This section compares that Plan with the ACF Roadmap and identifies gaps between them in terms of development pathways towards a high renewable net-zero power generation system.

### (a) Energy diversification

Table 2 compares key features of the ACF Roadmap [9] and the Generation Development Plan 2020 [24]. Carbon-free energy sources with potential that was reviewed and reported in the former were bioenergy (biomass, biogas, municipal solid waste), fuel cells (green hydrogen), geothermal, hydropower, nuclear, ocean thermal energy conversion (OTEC), solar photovoltaic (PV), wave, tidal & current and wind. For the latter, the RE sources considered were mainly biomass, biogas, hydropower and solar PV. The ACF Roadmap was developed based on the backcasting approach and so formulated the desired goal on an emission basis—to achieve net-zero CO_2_ emissions by 2050. It then suggested the requisite installed capacity of carbon-free energy sources for achieving that goal. By contrast, the Generation Development Plan was developed based on the forecasting approach and projected potential RE capacities to be installed in the next 5 and 15 years. Therefore, the targets of the Plan were formulated based on installed capacities, while at the same time ensuring that the NDC under the Paris Agreement will be achieved by 2030. For 2025, the Plan has a more ambitious RE target relative to the installed capacity that was anticipated by the Roadmap (table 2); however, both approaches expect a similar percentage of carbon-free energy in the total install capacity by 2035, at about 40%. In terms of diversification of the generation mix, the key distinction is that the Roadmap had more diverse carbon-free energy sources but limited the expansion of large hydropower, while RE in the Generation Development Plan is heavily dependent on hydropower and solar.

**Table 2.** Comparison of the ACF Roadmap and the Generation Development Plan 2020.

	ACF Roadmap [9]	Generation Development Plan 2020 [24]
present state (2018)	4.2% of RE in capacity mix, excluding large hydropower or 22.4% of RE in capacity mix, including large hydropower [3]	
scenario approach	backcasting	forecasting
emission target	net-zero CO_2_ emissions by 2050	reduce carbon emission intensity per GDP in 2030 by 45% relative to the 2005 level
estimated energy savings compared to business as usual	10% by 2050	8% by 2025
carbon-free resources	bioenergy (biomass, biogas, municipal solid waste), fuel cell (green hydrogen), geothermal, hydropower, nuclear, OTEC, solar PV, wave, tidal and current, wind	biomass, biogas, hydropower and solar PV
carbon-free energy in total installed capacity	19.6% by 2025	31% by 2025
	40% by 2035	40.5% by 2035

For the next roadmap development, indigenous RE sources other than hydropower and solar should be reviewed, including emerging technologies that will become mature over time. The Generation Development Plan projected RE contributions without breaking them down into types. The ACF Roadmap projected carbon-free energy contributions by source type, but their practical potential should still be assessed rigorously. A robust and transparent projection is crucial not only for providing figures and statistics, but also as a communication tool to present possibilities to decision makers and the public. Hence, an empirically rigorous study is critical for shaping the future energy system around high RE electrification.

### (b) Energy savings

EE is essential in decarbonizing the energy sector and complements electrification. Without EE improvement, it will be more difficult for RE to displace fossil fuels in electricity generation, particularly alongside growth in electricity demand. Nonetheless, at the time of development of the ACF Roadmap, there was no official energy savings target and thus the task force team estimated a conservative target to constitute 10% of electricity consumption reduction from business-as-usual (BAU) levels by 2050, with the reduction by 2025 estimated as about 3%. In the Generation Development Plan 2020, an 8% reduction of electricity consumption was anticipated by 2025; this is an aspirational target that the government set under the National Energy Efficiency Action Plan (NEEAP) (2016–2025) [25]. However, though the NEEAP clearly stated in the Executive Summary that it was ‘*devised for the country, including Sabah and Sarawak*’, the calculation of the 8% savings against BAU in table 3 of the document might not be referring to the whole nation; namely, the total electricity demand stated under the BAU level for 2016 was only 117 110 GWh. Meanwhile, the National Energy Balances indicate the total electricity demand for the whole nation has been increasing, with values of more than 117 000 GWh since 2013 [26]; in particular, demand was 132 199 GWh in 2015 [27]. Neither references nor further information were provided in the Plan in this regard. Consequently, due to the unclear scope of the target, we are unable to compare energy savings between the ACF Roadmap and the Generation Development Plan. We thus reserve arguments on whether savings potentials were overestimated or underestimated. Overestimation would result in more RE deployment being required to meet the target, because of the actual higher demand, while underestimation means the actual electricity demand would be lower than the deployment. In the case of the ACF Roadmap, underestimation would increase the likelihood of achieving net-zero by 2050. Because of these uncertainties, we are not able to estimate the gap between actual achievement and the projected scenarios.

We wish to highlight that at the early stage of a net-zero emissions pathway, EE measures are critical in minimizing energy demand growth and curbing emissions. EE measures are relatively less complex than RE deployment and can be scaled up rapidly, and there are technologies and measures that have been proven cost effective in improving EE in industry, buildings, appliances and transport. However, lack of enforcement could be a barrier in accelerating the implementation of EE measures in industry and commercial sectors [28]; as such, mandating energy performance monitoring and assessment should be considered under the provisions of the Efficient Management of Electrical Energy Regulations 2008 [29].

In addition, optimizing power plant performance is another potential area that could minimize energy losses and ensure efficient use of resources. The mode of operation, maintenance, ageing and degradation of power stations are factors that contribute to system performance; for instance, in 2014, scheduled maintenance contributed 1% of the average thermal efficiency improvement for coal thermal power plants [30]. Hence, the performance of power plants should be monitored on a regular basis and scheduled for maintenance or system optimization. The Energy Commission publishes annual reports on generation system performance. In 2018, there were coal and diesel power plants for which the average thermal efficiency dropped nearly 2% compared to 2017 [31]; these could present good opportunities for system optimization. A calculation based on a conservative assumption of 1% thermal efficiency improvement yielded an energy saving of about 349 ktoe, equivalent to the energy inputs for a coal power plant with capacity of about 189 MW. Reducing demand for fossil fuels like coal will result in emissions reduction. Hence, a comprehensive study on the potential energy savings from power plants and end-user consumption will be crucial as an input for the development of the next net-zero emissions roadmap. The synergy between RE and EE will offer a route for timely decarbonization in a cost-effective manner.

### (c) Deployment scales

Hydropower contributed the most to meeting the RE target under the Generation Development Plan. The current share of large hydropower is about 18% of total installed capacity [3]; this is more than half of the RE target, which was set as 31% by 2025. Notably, experts and stakeholders who were involved in the development process agreed that small-scale hydropower and roof-top solar PV would be prioritized over large hydropower and utility-scale solar plants. Large-scale plants were held back by concerns of land-use planning and environmental impact [32–38]. In addition, though a hydropower plant is able to operate 24 h a day throughout the year and with current technology can achieve an efficiency of up to 0.95 [9], the capacity factor for hydropower in Malaysia was only about 0.36 [3]. This was because large hydropower plants had been used as peaking plants, and the remaining capacity reserved to comply with a minimum reserve margin of 30% for the total national grid [9]. Experts and stakeholders agreed that operational capacity needs to be controlled to safeguard the environment, and if there is a need to increase hydropower capacity, the primary consideration should be to monitor actual operational performance and evaluate it in relation to the designed performance. This will give decision makers a better perspective on whether there is need for new hydropower plants. In this way, hydropower would maximize CO_2_ reduction by avoiding deforestation that contributes to CO_2_ emissions.

At the time of ACF Roadmap development, feed-in tariff (FiT) was the only existing policy mechanism for an RE installation of up to 30 MW. Net energy metering (NEM) was launched in 2016 to replace FiT for solar PV, while the second generation of NEM (NEM 2.0) was introduced in 2019, followed by the third generation in 2021. Under the NEM, excess energy produced from the installed solar PV system is exported to the national utility company, Tenaga National Berhad (TNB), on a ‘one-on-one’ offset basis. This scheme is applicable to all of TNB's consumers, including the domestic, commercial, industrial and agricultural sectors [39], and through it, the 500 MW quota allocation under NEM 2.0 was fully subscribed by 31 December 2020 [40]. Supported by other fiscal policies, the majority of that quota was taken by the industry sector. Companies from the industrial and commercial sectors that join in NEM are eligible for the green investment tax allowance (GITA) and the green income tax exemption (GITE), and further benefit from the Green Technology Financing Scheme 3.0 (GTFS3.0) for their green initiatives [41]. This scheme also provides a platform for residential consumers to participate in generating green energy.

**Figure 2.**
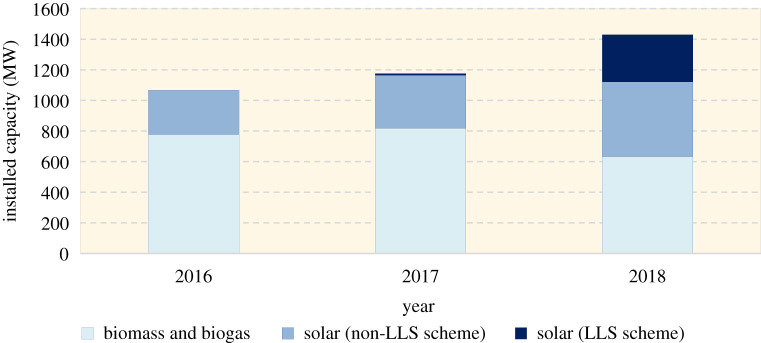
Renewable energy installed capacity, 2016–2018 [3,31,43–45]. (Online version in colour.)

Another innovation since the development of the ACF Roadmap is the large scale solar (LSS) scheme, which was introduced in 2016 [42]. LSS is a competitive bidding programme to drive down the levelized cost of energy (LCOE) for the development of large-scale solar PV plants with installed capacity ranging from 1 to 30 MW [42]. Following the implementation of the programme, 12 MW of total installed capacity was contributed to the grid in 2017, and then a total of 308.5 MW in 2018, an increase of nearly 26 times in just a year (figure 2). This added capacity accounted for about 39% of the total installed capacity from solar. In addition to the open tendering of the LSS having successfully lowered the LCOE of solar PV, technological advancement and the globally dropping cost of RE [12] have made the solar PV generation cost more competitive.

Thus, the impressive achievements of NEM 2.0 and LSS offer strong reasons to revise the fuels share in the generation mix for the ACF Roadmap, whereby the development of solar PV might need to consider potential beyond the roof-top scale in the next roadmap development. Nonetheless, while megaprojects might result in economic benefits, in some cases, they may not necessarily address energy poverty and improve livelihoods in their local communities. For instance, communities near the Bakun Dam are still not being supplied with treated water and electricity, even though the hydropower plant has been operating since 2011 [33,34,46,47]. This is similar to the case of a solar power plant in Rwanda [38]. Thus, large-scale RE power plants for high RE electrification need to be associated with a regulatory framework that protects the rights of the local communities. This will align with the Paris Agreement's objective that aims to strengthen the global response to the threat of climate change in the context of sustainable development and efforts to eradicate poverty [4].

### (d) Innovation in carbon-free energy technologies

At the time of the ACF Roadmap development (2015), there was an expected timeframe of about 30 years in which to achieve the net-zero vision. It was anticipated that carbon-free technologies would evolve during that period, such as through the advancement of existing technologies and innovation of new ones. Thus, the proposed generation mix was diversified into ten sources of fuels, i.e. nine carbon-free energy sources and a mix of fossil fuels. These sources included emerging technologies such as hydrogen and fuel cells, which would be key in offsetting fossil fuels emissions. These technologies were not popular at that time, but have been gaining more attention in recent years. Rahman & Wahid [48] analysed the potential and feasibility of green hydrogen in Malaysia and they argued that as an O&G producer, Malaysia has the advantage of using depleted natural gas reservoirs for hydrogen storage and delivering it through the existing gas network, thus requiring minimal retrofitting. Transporting hydrogen through pipelines will be 10–20 times cheaper and additionally reduce energy losses relative to electricity transmission by cable. Furthermore, green hydrogen can be shipped between Borneo and Peninsular Malaysia as gas in high-pressure containers, liquid in thermally insulated containers, processed methanol or ammonia or via a chemical carrier medium. Thus, geographical constraints will not hinder the market for the power industry or the hydrogen economy. Nevertheless, more support studies of hydrogen and fuel cells, ranging from pilot projects to scaling-up from a prototype to commercial scale, are vital in justifying these technologies as future fuels. Yue *et al*. [49] conducted a comprehensive review and concluded that hydrogen and fuel cells are critical in decarbonizing the energy system, including through complementing high uptakes of more variable renewable energy (VRE). However, fuel cells will need further improvement in several respects to become a cost-competitive technology in the power sector. Among the challenges they face are sustainability of water and rare materials consumption, the efficiency and durability of electrolyzers and fuel cell systems, and enabling policies for a hydrogen integrated energy system. Furthermore, IRENA recognized potential market opportunities for green hydrogen as feedstock for industrial processes and energy applications; also, it is the cheapest decarbonization option for hard-to-abate sectors such as the iron, steel, non-ferrous, petrochemical and cement industries [50]. This was not taken into account for the ACF Roadmap. Therefore, the projected RE contribution to electricity generation may need to consider whether RE-generated hydrogen will be used for other purposes besides the power sector. If so, a higher share of RE in electricity generation will be needed to meet hydrogen demand from other economic sectors.

Another non-mainstream technology introduced in the ACF Roadmap was OTEC. Follow-up studies have been conducted to estimate the OTEC resources at five selected sites in East Malaysia, located in deep ocean water (greater than 1000 m) with temperatures that ranged from 5 to 7°C. The results confirmed OTEC potential, with each studied site indicated to have a potential of at least 2.5 MW and up to 10 MW [51]. It is worth mentioning that one of OTEC's roles in the ACF Roadmap was to generate hydrogen offshore; the power generated from OTEC can be used to produce methanol and ammonia, and those hydrogen-enriched chemicals can be directly fed to fuel cells for power generation. In addition, Banerjee *et al*. [52] argued that advancement in OTEC technology would reduce electricity production cost through tapping the by-products of OTEC. These prospects support our proposal of a generation mix in which fuel cells and OTEC constitute large portions.

**Figure3.**
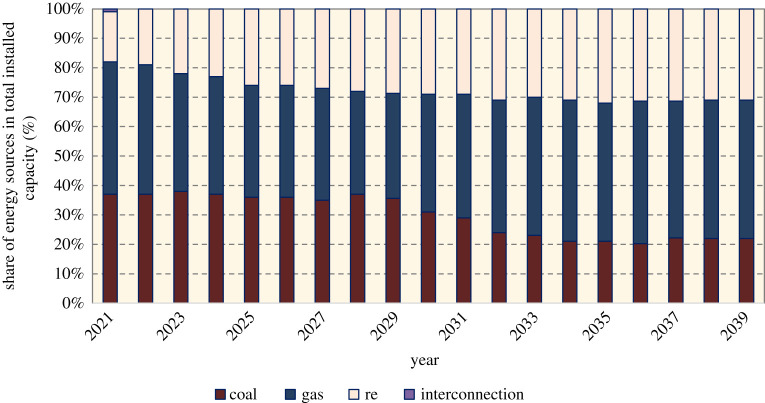
Share of energy sources in total installed capacity for Peninsular Malaysia as proposed in the Generation Development Plan 2020 [24]. (Online version in colour.)

**Figure 4.**
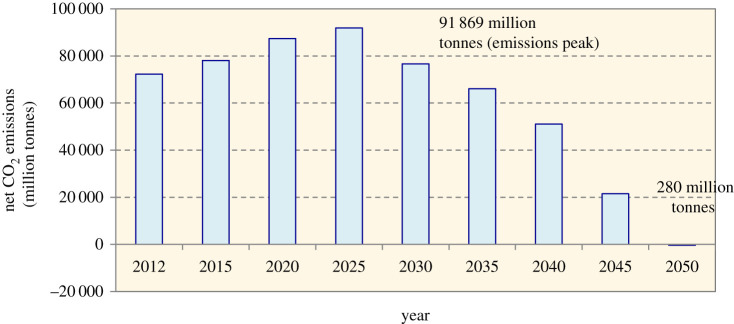
Net CO_2_ emissions (2012–2015) as estimated by the ACF Roadmap [9], in which emissions would peak around 2025 and gradually decrease to net-zero or below relative to the amount emitted to the atmosphere (negative emissions) by 2050. (Online version in colour.)

Nevertheless, when planning for large-scale deployments of emerging technologies, the urgency of addressing emissions reduction needs to be taken into consideration. Promising carbon-free energy methods that already have technology readiness may need to be prioritized while emerging technologies are included in the long-term plan. The ACF Roadmap anticipated OTEC and fuel cells would come into place around 2020 (figure 3); however, as of mid-2021, there is no sign that these technologies could be integrated into the power grid. Meanwhile, the Generation Development Plan did not segregate RE share by type (figure 4). Information on the types of emerging technologies that are considered for future-oriented policies and plans is critical, as every aspect of technology development from innovation to scaling-up needs policy enablers to support large-scale deployment and infrastructure planning. This process could be complex and might involve cross-sectoral integration (e.g. the hydrogen economy). And so, it is important to have a transparent development plan that prepares all stakeholders to start planning for deployment of such technologies and meeting the net-zero aspiration on time. Furthermore, realizing a high contribution of renewables in electricity generation will require grid upgrades. Global trends in boosting RE include innovations such as a decentralized power system, peer-to-peer (P2P) electricity trading and community-based RE. Additionally, shifting to electrification in areas such as vehicles may result in higher electricity demand. High RE with energy storage would be key in securing a low-carbon electricity supply. None of these topics were addressed in either the Roadmap or the Plan, and should be considered with clear strategies in the next plan.

### (e) Emissions of carbon-based fuels in the generation mix

**Figure 5.**
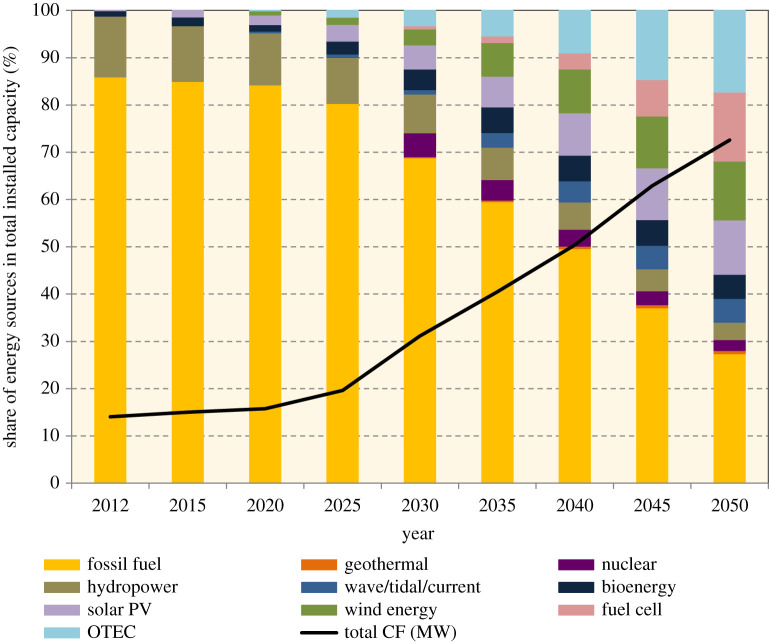
Share of energy sources in total installed capacity as proposed by the ACF Roadmap [9]. (Online version in colour.)

The ACF Roadmap found that the proposed generation mix will lead the power sector to reach an emission peak by as soon as around 2025 (figure 5). However, the ACF Roadmap did not segregate fossil fuel sources but rather used the overall average emission factor in calculating CO_2_ emission, assuming that 1 MWh of generated electricity is equivalent to 0.63 tonnes of CO_2_ emission. This emission factor referenced the calculation that was used in the National Renewable Energy Policy and Action Plan (NREPAP) [21]. In addition, in the third Biennial Update Report (BUR) [7] submitted to the United Nations Framework Convention on Climate Change (UNFCCC), Malaysia estimated the 2016 grid electricity emissions using three different emission factors for Peninsular Malaysia, Sabah and Sarawak, which were 0.639 tonnes CO_2_/MWh, 0.512 tonnes CO_2_/MWh and 0.249 tonnes CO_2_/MWh, respectively. Given this, the estimated equivalence that was used in the ACF Roadmap (0.63 tonnes CO_2_/MWh) probably is valid up to 2016. Nonetheless, different types of fossil fuels will result in different amounts of emissions, and when the electricity share of RE is increasing, the emission factor will be lower. In the ACF Roadmap, a constant emission factor was used for estimating emissions avoidance throughout the net-zero pathway; this may result in overestimation of actual emissions from fossil fuels, and hence require a higher RE capacity to offset those emissions. Meanwhile, the Generation Development Plan showed the alignment of the generation mix in achieving the country's NDC, thereby to reduce its 2030 economy-wide carbon intensity (against GDP) by 45% relative to the 2005 level [6]. However, the Plan included no information on projected GDP and the amount of emissions avoidance. We argue that the transparency and accuracy of emissions calculations may be enhanced by, instead of a compound emission factor, using individual emission factors for each of the different types of fuels. Doing so is particularly relevant when the desired goal is an emission-based target that was formulated to attain carbon neutrality in the power sector.

## 4. Potential future research

Based on the above review, we argue that the ACF Roadmap is still relevant to the net-zero vision. Nonetheless, we have also identified gaps that merit further research and improvement. Accordingly, we put forward the following potential research suggestions, which mainly aim to address the knowledge gaps that we have identified from the present review; however, there might be other critical research areas that go beyond the scope of this paper.

— *An integrated approach for developing a net-zero emissions roadmap*. The ACF Roadmap was a simplified version in which scenarios were developed through stakeholder consultation. Constructing a net-zero model via an integrated approach may provide more reliable and constructive recommendations. In particular, a model that integrates forecasting and backcasting approaches and is assisted through a stakeholder consultation process will provide a more feasible transition scenario. Stakeholder participation will create a sense of ownership and enhance the model's credibility among stakeholders in realizing the long-term goal.
— *Energy savings from EE measures.* Studies on potential energy savings from all sectors are crucial. Energy outlook and energy-saving potential are important information in planning for a net-zero pathway, and minimizing energy demand will ensure a cost-effective system with high RE electrification. As such, it is important to institutionalize baseline performance indicators for the end-user economic sectors as well as the power plants. Subsequently, a workable auditing system may need to be developed in order to facilitate these performance improvement initiatives. These developments will enhance the availability and reliability of the information on energy demand and energy-saving projections, and so contribute to ensuring a resource-efficient power system with high penetration of RE.
— *Policy transparency, clarity and consistency.* As discussed earlier, when reviewing energy savings targets, we found the NEEAP to be inconsistent in defining scope [25]. A similar issue was also observed in the Generation Development Plan 2020. For instance, paragraph 1.2 defined the target of 31% RE as for Peninsular Malaysia, while figures 5 and 7 of the document stated targets of 31% for Malaysia and 26% for Peninsular Malaysia [24]. Policy documents such as the NEEAP and the Generation Development Plan are key references for modellers when developing outlooks, which subsequently provide guides for decision makers in shaping future policies and directions. Furthermore, the extent to which the general populace has been informed about the national aspirational targets and the role of the media in communicating such information could be critical. Therefore, studies related to policy transparency would be crucial in strengthening the policy analysis.
— *Emerging technologies in the local context.* Scaling-up of RE integration is essential to achieve a net-zero emissions power system. To support such scaling, more studies in the local context need to be carried out to address the intermittency of VRE and the challenges of ensuring the stability of transmission and distribution grid networks. Among the potential research areas are the decentralization and digitalization of energy systems, including P2P trading and battery and energy storage [53–55]. Beyond RE integration, supply-side EE should not be neglected; for example, optimizing and improving the efficiency of existing power plants may avoid the construction of new power plants [28]. Moreover, long-term planning will need to consider future electricity demands associated with shifting of market demand from conventional fuels to electrification technologies, e.g. electric vehicles. Though the net-zero target concerns the next 20 or 30 years, building or upgrading infrastructure and regulating law and legislation are all lengthy processes. Backcasting signals the urgency of needed actions, whereas knowledge of the science and technology to be used in addressing the technical challenges will justify the readiness of such interventions.
— *Balancing the energy trilemma and just transition.* As expressed in the Generation Development Plan 2020, balancing the energy trilemma (energy security, affordability and sustainability) is a key policy objective. It is commendable that reducing dependency on coal is one of the aspirations under the element of sustainability, yet new coal power plants totalling 2800 MW will be added to the grid between 2031 and 2037 [24]. There was no explanation given concerning the role of coal in the context of the energy trilemma. On the other hand, in the context of RE, the Bakun hydropower plant has been a case study in energy justice issues [33,34,46,47]. As such, when accelerating the low-carbon transition through large-scale RE deployment, it is also necessary to ensure the transition will bring inclusive growth. Shaping the pathways to the net-zero vision should not contradict poverty alleviation and sustainable development. Studies on the social and economic impacts of infrastructure development could provide information beyond the mandated requirements of the Environmental Impact Assessment [35,56]. Such research findings could assist in decision-making regarding fuel types and deployment scales [57].

Given the gaps that were identified in this present review, we recommend the development of a revised carbon-free energy roadmap. The ACF Roadmap could serve as the foundation of this roadmap and be improved upon with the most current energy data. Creating a revised version also offers an opportunity to update the generation mix and action plans to align with trends in technology development and the latest technological breakthroughs. As of this writing, the government has yet to announce its aspirations concerning the net-zero vision or an absolute emissions reduction target; as such, a revised roadmap could be used as a tool for communicating the net-zero vision to policy-makers. Some researchers have reported that Malaysia's energy policy is moving towards a low-carbon transition, but more support is needed for accelerating carbon-free energy deployment [48]. A net-zero emissions vision guided by a roadmap may effectively motivate and catalyse carbon-free energy deployments.

## Data Availability

All the data are based on publicly available documents, which the data sources were cited in the manuscript.
